# Disparities in Hospital-Reported Breast Milk Use in Neonatal Intensive Care Units — United States, 2015

**DOI:** 10.15585/mmwr.mm6648a1

**Published:** 2017-12-08

**Authors:** Ellen O. Boundy, Cria G. Perrine, Jennifer M. Nelson, Heather C. Hamner

**Affiliations:** ^1^Epidemic Intelligence Service, CDC; ^2^Division of Nutrition, Physical Activity, and Obesity, National Center for Chronic Disease Prevention and Health Promotion, CDC.

Breast milk is the recommended nutrition for infants. For preterm infants, when mother’s milk is not available, pasteurized donor milk is recommended (*1*). Non-Hispanic black mothers are at increased risk for having a preterm birth and for not breastfeeding (*2*,*3*); however, it is not known whether demographic disparities exist in the use of breast milk in neonatal intensive care units (NICUs). Data from CDC’s 2015 Maternity Practices in Infant Nutrition and Care (mPINC) survey, which does not collect patient-level demographics, were linked to the 2011–2015 U.S. Census Bureau’s American Community Survey (ACS)* to examine use of breast milk in NICUs based on demographic makeup of the hospital’s postal code area. Among U.S. hospitals with a NICU, the use of mother’s own milk and donor milk were examined by the percentage of non-Hispanic black (black) residents in the hospital postal code area, categorized as being above or below the national average (12.3%). In postal codes with >12.3% black residents, 48.9% of hospitals reported using mothers’ own milk in ≥75% of infants in the NICU, and 38.0% reported not using donor milk, compared with 63.8% and 29.6% of hospitals, respectively, in postal codes with ≤12.3% black residents. Further investigation is needed to understand variations in breast milk use in NICUs. Targeted efforts to increase breast milk use in hospitals located in postal codes where the percentage of black mothers is above the national average might help ensure more equitable access to breast milk for preterm and other high-risk infants.

The American Academy of Pediatrics (AAP) recommends that infants receive breast milk. In addition to the nutritional benefits of breast milk, consumption of breast milk by preterm infants is associated with lower rates of sepsis and necrotizing enterocolitis, and a number of other improved health outcomes (*1*). When mother’s own milk is contraindicated or insufficient, pasteurized donor milk is recommended (*4*). Black mothers are at increased risk for preterm birth and delivering a low birthweight infant and have lower rates of breastfeeding initiation and duration than do white and Hispanic mothers (*2*,*3*). Mothers of infants in the NICU often face challenges with breastfeeding because of their infants’ health conditions as well as being separated from their infants. The use of donor breast milk for high-risk infants is increasing, but access continues to be limited as hospital demand outpaces milk bank supply (*4*,*5*). Little is known about disparities in the use of breast milk for infants hospitalized in NICUs.

CDC’s mPINC survey is a census of facilities providing maternity care in the United States and territories (*6*). The survey is completed by the person or persons who are most knowledgeable about the facility’s practices related to infant nutrition. Information collected included facility characteristics, including hospital type; whether the facility is a teaching hospital; size (births per year); and neonatal care unit level (classified as level III or IV based on their ability to provide risk-appropriate subspecialty intensive care). Hospitals with a NICU report the approximate percentage of infants in the NICU routinely receiving mother’s own breast milk and banked donor breast milk. Because patient-level demographics are not collected as part of the mPINC, neighborhood-level data were obtained from the ACS to explore potential racial disparities. ACS is an ongoing survey of demographic, social, and housing characteristics, with postal code–level race data reported in 5-year estimates. Data from the 2015 mPINC were linked to 2011–2015 ACS data by hospital postal code. In 2015, the mPINC response rate was 82%, and included 2,582 participating facilities. Among 654 hospitals with a NICU, 602 (92.0%) had postal code–level race data available in ACS, including 576 (95.7%) and 568 (94.4%) that had data on mother’s own milk use and donor milk use, respectively. Hospitals were categorized as being in a postal code where the percentage of black residents was >12.3% (the national average) or ≤12.3%. No accepted cut-points exist for the prevalence of infants in the NICU receiving breast milk; therefore, receipt of breast milk was grouped into four categories at 25% intervals to illustrate the distribution of use across hospitals.

Because data were skewed, the median prevalence and interquartile range (IQR) of infants routinely receiving mother’s own and donor milk was calculated across hospitals, with stratification by the percentage of black residents in the hospital postal code above or below the national average. Chi-square, Fisher’s Exact, and, for continuous variables, Wilcoxon Rank-Sum tests were performed using statistical software.

Among 602 hospitals with NICUs, 222 (36.9%) were located in postal codes where the percentage of black residents exceeded the national average (Table 1). Overall, 145 of 580 (25.0%) hospitals were teaching hospitals, and 86 of 579 (14.9%) were government-run; in postal codes with higher percentages of black residents, the percentage of teaching hospitals (32.4%) and of government-run hospitals (20.5%) was higher than in postal codes with lower percentages of black residents (20.6% and 11.5%, respectively). NICU level and facility size were similar in hospitals in postal codes with high and low percentages of black residents.

**TABLE 1:** Characteristics of hospitals with a neonatal intensive care unit, by racial composition of hospital postal code area — United States, 2015

Hospital characteristic	All hospitals (n = 602), no. (%)^*^	Percentage of non-Hispanic black residents in hospital postal code area, no. (%)		p-value^†^
		≤12.3% (n = 380)	>12.3% (n = 222)	
**Neonatal intensive care unit level^§^**				0.24
III	525 (87.2)	336 (88.4)	189 (85.1)	
IV	77 (12.8)	44 (11.6)	33 (14.9)	
**Hospital type^¶^**				0.01
Government	86 (14.9)	42 (11.5)	44 (20.5)	
Nonprofit	406 (70.1)	264 (72.5)	142 (66.0)	
Private	87 (15.0)	58 (15.9)	29 (13.5)	
**Teaching hospital**				<0.01
Yes	145 (25.0)	75 (20.6)	70 (32.4)	
No	435 (75.0)	289 (79.4)	146 (67.6)	
**Facility size (no. of births in past year)**				0.45
1–499	9 (1.5)	6 (1.6)	3 (1.4)	
500–999	38 (6.3)	28 (7.4)	10 (4.5)	
1000–1999	166 (27.6)	106 (27.9)	60 (27.0)	
2000–4999	344 (57.1)	216 (56.8)	128 (57.7)	
≥5000	45 (7.5)	24 (6.3)	21 (9.5)	

* Total number does not sum to 602 for hospital type and teaching status because of missing values (n = 22).

^†^ Chi-square test or Fisher’s Exact test if one or more cells with expected count <5.

^§^ Level III indicates facilities with capability to care for infants born before 32 weeks’ gestational age and weighing <1500 g and infants born at all gestational ages and birthweights with critical illnesses, with availability of a range of pediatric subspecialists; level IV indicates regional neonatal intensive care units with all level III capabilities, plus availability of pediatric surgical subspecialists.

^¶^ Military hospital data excluded in stratification by hospital type because of the small number of facilities, but are included in all other analyses.

Across all hospitals, the median estimated prevalence of infants in NICUs receiving mother’s own milk was 75.0% (IQR = 60.0%–86.0%); the percentage was higher in NICUs in postal codes with lower percentages of black residents (80.0%) than in those in postal codes with higher percentages of black residents (72.0%) (p<0.01) (Table 2). The median prevalence of infants receiving banked donor breast milk across all NICUs was 10.0% (IQR = 0%–20.0%); the percentage was higher in NICUs in postal codes with lower percentages of black residents (10.0%) than in NICUs in postal codes with higher percentages of black residents (5.0%) (p = 0.04) (Table 2).

**TABLE 2:** Percentage of infants routinely receiving mother’s own breast milk and banked donor breast milk in neonatal intensive care units, by racial composition of hospital postal code area — United States, 2015

Source of breast milk	No.	Median (interquartile range), (%)	Range, %	p-value*
**Mother’s own breast milk**				
Total (all hospitals)	576	75.0 (60.0–86.0)	0–100	<0.01
Percentage of non-Hispanic black residents in hospital postal code area				
Low^†^	359	80.0 (65.0–90.0)	0–100	
High	217	72.0 (60.0–85.0)	2.0–100	
**Banked donor breast milk**				
Total (all hospitals)	568	10.0 (0–20.0)	0–100	0.04
Percentage of non-Hispanic black residents in hospital postal code area				
Low	352	10.0 (0–20.0)	0–100	
High	216	5.0 (0–20.0)	0–100	

* Wilcoxon Rank-Sum test.

^†^ Low: ≤12.3% (national average); high: >12.3%.

Less than half (48.9%) of hospitals in postal codes with higher percentages of black residents reported that ≥75% of infants in the NICU received mother’s own breast milk, compared with 63.8% of NICUs in postal codes with lower percentages of black residents (Figure). Similarly, 38.0% of hospitals in postal codes with higher percentages of black residents reported that no infants in the NICU received donor breast milk, and 5.1% reported that at least half of infants in the NICU received donor breast milk, compared with 29.6% and 11.4%, respectively, of NICUs in postal codes with lower percentages of black residents (Figure).

**FIGURE:**
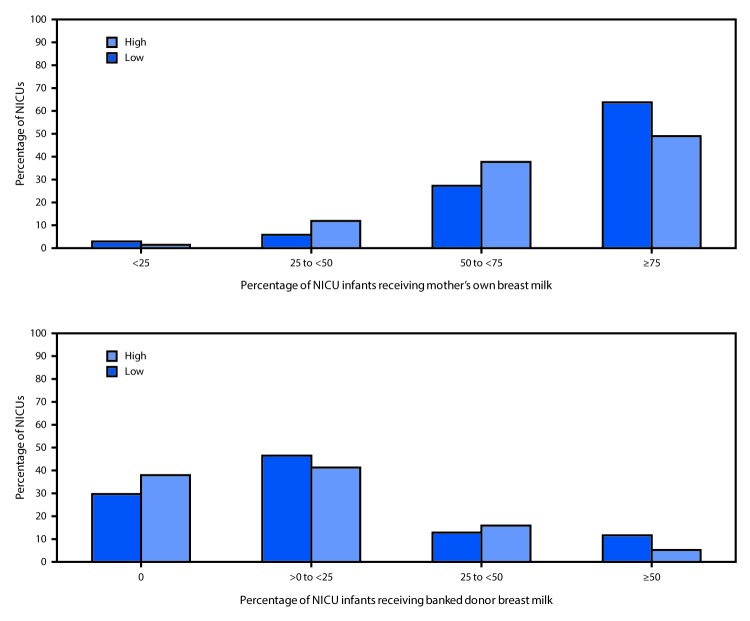
Percentage of infants in neonatal intensive care units (NICUs) receiving mother’s own breast milk or banked donor breast milk, by racial composition of hospital postal code area* — United States, 2015 * Percentage of non-Hispanic black residents in hospital postal code area. Low: ≤12.3% (national average); high: >12.3%.

## Discussion

The use of both mother’s own and donor breast milk in NICUs was lower in hospitals located in postal codes with higher percentages of black residents than those in areas with lower percentages of black residents. This suggests that disparities exist in the provision of breast milk for high-risk infants by community or hospital characteristics despite breastfeeding being the optimal form of nutrition in their first days of life (*1*).

Differences in breast milk use in NICUs by racial composition of the surrounding community might be related to a range of factors, similar to those that have been found to affect breastfeeding rates overall. These include variations in health care personnel support, hospital policies and practices, mothers’ knowledge and access to information, and community-level support for breastfeeding (*7*,*8*). Donor milk use might also be affected by hospital proximity to milk banks, state regulations and hospital policies related to the provision of donor milk, and insurance reimbursement (*4*). There are currently 23 nonprofit milk banks accredited by the Human Milk Banking Association of North America, 10 of which are located in postal codes with a percentage of black residents >12.3%, as well as other commercial for-profit milk banks across the United States.^†^

The findings in this report are subject to at least five limitations. First, neighborhood demographics were used as a proxy for the racial makeup of the hospital’s patient population. It was assumed that women tend to use hospitals in their postal code of residence, which is not always the case. If a significant proportion of mothers choose to seek care or are transferred to hospitals in postal codes of a different racial makeup than their own, then it is possible that our results might be biased. However, there was no statistical difference in the level of care provided by NICUs by percentage of black residents. In addition, the sample was limited to level III and IV NICUs, which provide care for high-risk patients, thereby attempting to reduce this potential bias. Second, the reported percentage of infants receiving breast milk might be inaccurate, but this is not likely to differ by racial make-up of the hospital community. Third, although AAP recommends that all infants receive mother’s own milk unless it is unavailable or contraindicated (*1*), the percentage of high-risk infants who should receive donor milk is unknown, making interpretation of these results challenging. Fourth, mPINC does not capture data on NICUs in hospitals that do not perform deliveries, such as some children’s hospitals. Finally, nonresponse bias was also possible.

AAP recommends breast milk as the primary source of nutrition for infants, and supports equal access to donor breast milk based on medical necessity for all high-risk infants when mother’s milk is unavailable (*1*,*4*). The 2011 Surgeon General’s Call to Action to Support Breastfeeding recommends that stakeholders “identify and address obstacles to greater availability of safe banked donor milk for fragile infants” (*9*). Interventions aimed at increasing the use of breast milk in NICUs among hospitals serving higher percentage black patient populations might help reduce some of the disparities observed in this analysis. Health care providers can play a role in facilitating initiation of breastfeeding or breast milk expression after birth. Hospitals can ensure that policies and staff member training are in place to support provision of breast milk specific to high-risk infants. Safe and equitable access to milk from donor banks is also a factor in ensuring that all high-risk infants receive optimal nutrition. Understanding policies and practices at hospitals with higher breast milk use in the NICU might help inform interventions to increase its use in other facilities. Further investigation into cultural and community practices and preferences related to breast milk might also help in understanding differences in its use.

**Summary**

**What is already known about this topic?**

Breast milk is the recommended nutrition for infants, and is particularly beneficial for preterm infants. Non-Hispanic black mothers are at increased risk for preterm birth, and also have lower breastfeeding rates. Some data suggest there might be limited access to donor milk, which is recommended for preterm and other high-risk infants when mother’s milk is unavailable.

**What is added by this report?**

Data from the 2015 Maternity Practices in Infant Nutrition and Care (mPINC) survey of all U.S. maternity facilities, linked with postal code–level race data from the U.S. Census, found that hospitals in areas with higher percentages of black residents reported lower percentages of infants in the neonatal intensive care unit (NICU) routinely receiving mother’s own breast milk (median = 72.0%) or banked donor breast milk (median = 5.0%) than did hospitals in areas with lower percentages of black residents (median = 80.0% and 10.0%, respectively).

**What are the implications for public health practice?**

Targeted interventions among hospitals in areas serving a higher proportion of non-Hispanic black residents might help ensure more equitable access to breast milk for all high-risk infants. Further investigation is needed to understand factors affecting variations in breast milk use in NICUs.

## Footnotes

* https://www.census.gov/programs-surveys/acs/.

† https://www.hmbana.org.
